# Translation, cultural adaptation, and psychometric testing of the Supportive care needs survey for partners and caregivers for Swedish family members of persons diagnosed with colorectal cancer

**DOI:** 10.1186/s41687-023-00636-1

**Published:** 2023-10-11

**Authors:** Maria Samuelsson, Anne Wennick, Mariette Bengtsson, Marie-Louise Lydrup, Jenny Jakobsson

**Affiliations:** 1 Faculty of Health and Society, Department of Care Science, Malmö University, Jan Waldenströms gata 25, 205 06 Malmö, Sweden; 2 Department of Pediatrics, Skåne University Hospital, Malmö, Sweden; 3 Department of Surgery and Gastroenterology, Skåne University Hospital, Malmö, Sweden

**Keywords:** Colorectal cancer, Caregivers, Cognitive interviews, Family members, Psychometrics, Supportive care needs, Translation, Unmet needs, Validity, Questionnaire

## Abstract

**Background:**

Colorectal cancer is the third most common cancer diagnosis globally and is increasing in both incidence and prevalence. Despite evidence showing that family members of persons diagnosed with cancer have supportive care needs, no validated questionnaire measuring the needs of family members of persons diagnosed with CRC exists in Swedish. Thus, the objective of the present study was to translate, culturally adapt, and evaluate the psychometric properties the Supportive Care Needs Survey – Partners and Caregivers 45.

**Methods:**

The translation and cultural adaptation followed a systematic yet iterative process. Firstly, the questionnaire was translated using a forward–backward approach. Secondly, face and content validity and comprehensibility were evaluated by two expert panels of colorectal cancer specialist nurses and family members, respectively. Lastly, the psychometric properties, validity, and reliability of the translated questionnaire were evaluated among 45 Swedish family members of persons diagnosed with colorectal cancer.

**Results:**

The face, content, and construct validity of the translated questionnaire were evaluated as satisfying. Moreover, psychometric evaluations showed high data quality and satisfactory internal consistency. However, the results also revealed unsolved issues regarding relevance, targeting, and internal consistency, as well as a probable scaling failure.

**Conclusion:**

The translated and adapted questionnaire can be used to identify family members unmet needs of support throughout the colorectal cancer trajectory. The questionnaire showed promising validity and reliability in the target population. However, it needs to be further evaluated in a larger sample, preferably involving factor analysis and stability over time.

## Abstract (plain language summary)

Colorectal cancer is the third most common cancer diagnose globally. At times of cancer, also the health and wellbeing of the surrounding family members is negatively affected. As a result, family members of persons diagnosed with cancer report that they too need support. Still, no validated questionnaire that enable measurement of family members needs of support throughout the colorectal cancer trajectory existed in Swedish. Thus, the present study undertook the process of translation of a questionnaire from English to Swedish. Thereto, evaluated it among Swedish family members of persons diagnosed with colorectal cancer. The evaluation showed a successful translation and the translated questionnaire appeared reliable and useful for measuring the family members´ needs of support throughout the colorectal cancer trajectory. However, it requires further evaluation.

## Introduction

Family members of persons diagnosed with cancer, such as colorectal cancer (CRC), report unmet emotional, informational, relational, spiritual, and practical needs of support [1–5]. Supportive care needs are explained as connected to the shifting phases of a certain cancer trajectory [6, 7]. As a result, a homogenous support may be insufficient to meet family members´ needs. Instead, support should be designed in coherence with needs related to a specific cancer diagnosis [6–9]. Despite CRC being the fourth most common cancer diagnosis in Sweden, no validated questionnaire allowing the identification of family members’ supportive care needs exists in Swedish. Hence, a literature review was conducted to identify questionnaires potential for translation and cultural adaptation. The Supportive Care Needs Survey – Partners and Caregivers (SCNS-P&C45), originally developed by Girgis et al. [10], met the desired criterions; applicable across the CRC trajectory toward survival, designed to capture the width of potential supportive care needs, acceptable length and with satisfactory psychometric properties. This study employs Wright and Leahey’s [11] definition of “family member”, stating that the family is defined by the persons themselves. Hence, the family may involve next of kin and friends as well as biologically related persons.

SCNS-P&C45 consist of 45 items that measure family members’ unmet needs of support over the past month on a five-point scale. The response scale distinguishes between no needs and unmet needs where no needs are identified by merging response alternative 1 (not applicable) and 2 (fulfilled needs) while unmet needs are identified by aggregating response alternatives 3, 4 and 5 (low, moderate, and high). The total score ranges 45–225, a higher score indicating more needs. SCNS-P&C45 [10] was developed in Australia based on a literature review of the main supportive care needs of family members of persons diagnosed with cancer [1], an examination of existing tools assessing family members’ unmet needs, and adaptation of the items from the Supportive Care Needs Survey for persons diagnosed with cancer by [13, 14]. The development of items involved evaluating face and content validity by members of the public, experts in psycho-oncology, and family members of cancer survivors [10]. The five-point response scale was originally modelled on the Supportive Care Needs Survey for persons diagnosed with cancer [13, 14]. The psychometric properties of the SCNS-P&C45 were evaluated among family members. Principal factor analysis identified four underlying domains. Construct validity of the domains was supported. In addition, the questionnaire demonstrated satisfactory internal consistency, with Cronbach’s alpha ranging from 0.88 to 0.94 for the four domains (ibid).

The SCNS-P&C45 has been translated to several languages and tested for its psychometric properties. It is available in French [3], German [2], Dutch [15], Turkish [4], Chinese [5], and Thai [16]. In addition, the different versions of SCNS-P&C45 have been reported as promising for clinical use in groups of family members of persons with varying cancer diagnoses [2–5, 10] and in groups with a specific diagnosis (breast cancer and cholangiocarcinoma) [15, 16]. Thus, the current study aimed to translate, culturally adapt, and evaluate the psychometric properties of the SCNS-P&C45.

## Material and method

**Fig. 1:**
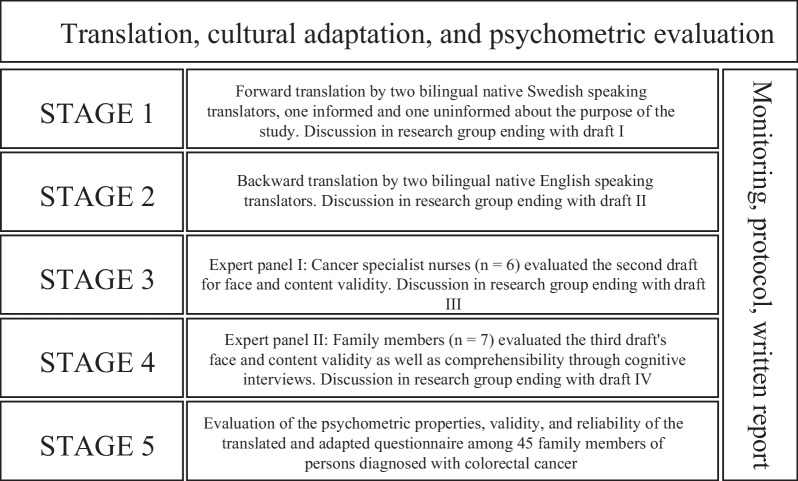
Process of translation, cultural adaptation, and psychometric evaluation

This study involved a translation, cultural adaptation, and psychometric evaluation following the COSMIN checklist [17]. The process was conducted in five stages, as illustrated in Fig. 1. A forward–backward translation was conducted through an iterative process, influenced by Beaton et al. [18]. Prior to translation, permission was obtained from Professor Girgis, the first author of the original SCNS-P&C45. The face and content validity and the comprehensibility of the translated questionnaire were then evaluated. Lastly, the psychometric properties, validity, and reliability of the translated and adapted questionnaire were evaluated in a pre-test.

### Translation

In stages one and two, the original questionnaire was translated. First, a translation from English to Swedish was conducted independently by two authorized translators. During translation, the translators wrote a report on perceived difficulties or conceptual uncertainties. The translation, with its written report, was then discussed by the research group (the authors – who are experienced in psychometric testing, supportive care for families, and clinical CRC care (as registered nurses and physician)) – until consensus on a first draft was reached. In the second stage, a backtranslation of the first draft, along with documentation of experienced difficulties, was conducted independently by yet another two authorized translators. Finally, the research group again reviewed and discussed all versions and written reports until consensus was achieved on a second draft.

### Face and content validity

In stages three and four, the face and content validity of the second draft was evaluated per item and scale by two expert panels including CRC specialist nurses and family members of persons diagnosed with CRC, respectively. The number of participants was based on Lynn’s [19] recommendation to use an expert panel including 3–10 persons.

For face validity, the CRC specialist nurses evaluated the translated questionnaire through a written report, whilst family members performed a verbal evaluation. Content validity was evaluated by the expert panel of CRC specialist nurses using the content validity index (CVI) and by the expert panel of family members through cognitive interviews. For CVI, the CRC specialist nurses were provided the translated questionnaire and asked to assess each item and the scale as a whole for relevance on a scale ranging from 1 = *not relevant* to 4 = *highly relevant*.

Cognitive interviews, involving the think-aloud method as described by Willis [20], were conducted by the first author who has experience of qualitative interviewing. The interviews were conducted to evaluate comprehensibility and whether the translated questions functioned as intended. Thus, family members (one partner and six adult children aged 33–70 years, all of which with higher education) were recruited through snowball sampling via a governmental organization for cancer care – Regional Cancer Centre South. During the interviews, participants were asked to read each item and response category out loud and explain how it was understood. Further, they were asked to exemplify a response. The interviews were conducted individually in two rounds, including five persons in the first round and two in the second round. The interviews were audio-recorded and transcribed in a protocol. The number of participants was guided by Lynn’s [19] recommendation and Willis’ [20] endorsement to recruit until no substantial new information emerged. After the first round, the interview transcripts were reviewed by the whole research group, which lead to conceptual and semantic revisions. The second round of interviews revealed no further uncertainties. Consequently, in accordance with Willis [20], it was appraised that the identified problems had been resolved and interviewing could stop.

### Evaluation of the Swedish SCNS-P&C45s’ psychometric properties, validity, and reliability

In the fifth stage, the Swedish SCNS-P&C45 (SCNS-P&C45-S) was evaluated in a pre-test including 45 Swedish family members of persons diagnosed with CRC. Family members were recruited consecutively by health care professionals from four surgical outpatient clinics in Sweden. To be eligible for recruitment, potential participants had to be identified by the person diagnosed with CRC as family members, have contact (direct or indirect via the patient) with an outpatient CRC clinic, and be able to read and understand Swedish. Exclusion criteria were family members of a person receiving or expected to require palliative care, since the questionnaire is to be used among family members of persons with expected survival. Sample size was based on recommendations by Beaton et al. [18] and Coenen et al. [21]. The participants were informed about the purpose and procedure of the study and provided a letter of information via a gatekeeper at the clinic or, since family members rarely visited the clinic, via the person diagnosed with CRC. Attached to the letter of information was the SCNS-P&C45-S, a consent form, sociodemographic questions, a pre-paid reply envelope and, the Hospital Anxiety and Depression Scale (HADS) to evaluate construct validity.

### Data analysis

#### Face and content validity

Data from the two expert panels were reviewed regarding face validity. Item-content validity (I-CVI), average scale content validity (S-CVI/Ave), and universal agreement scale content validity (S-CVI/UA) were analysed in accordance with Polit and Beck [22]. Using six experts, the threshold of I-CVI should be ≥ 0.83 and of S-CVI ≥ 0.80 [22]. The data from the cognitive interviews were reviewed to identify semantic and conceptual issues. Subsequently, they were discussed and revised by the research group in relation to Swedish CRC care and the conceptual ideas underpinning the original SCNS-P&C45.

#### Psychometric evaluation, validity, and reliability

Psychometric analyses of the SCNS-P&C45-S were performed using IBM SPSS Statistics for Windows, Version 28.0. (Armonk, NY: IBM Corp). Descriptive statistics (i.e., means and standard deviations [SD]) were used to evaluate data quality, scaling assumptions, and targeting. Construct validity was evaluated through convergent validity and reliability through internal consistency. Analyses for convergent and internal validity as well as internal consistency of SCNS-P&C45-S domains were based on a four-point scale, as proposed by Girgis et al. [10]. Accordingly, the response categories 1 and 2 (*not applicable* and *satisfied*) were regrouped to 1, *No need*. However, descriptive statistics of the items and response options were based on the five-point scale, since it enabled distinguishing items assessed as “not relevant” and conducting a more thorough examination of response options.

#### Data quality

Data quality was evaluated using the proportion of missing data per item and percentage of computable scale scores since, according to Hobart et al. [23], this reflects the participants’ understanding and acceptance of a measure. As recommended by Hobart et al. [23], data quality was determined to be high if the proportion of missing data per item was low, with a threshold of < 10% considered as acceptable. Further, participants leaving more than 50% of questions blank were excluded from further analysis, whilst remaining missing items were imputed based on the respondents’ mean.

#### Scaling assumptions

In Likert-based scales, items can be summed if they have roughly equivalent means and standard deviations [23]. In addition, within a domain, all items should contribute equally and substantially (*r* ≥ 0.30) to the total score. Accordingly, the item response distributions of the SCNS-P&C45-S domains were reviewed and item-total correlations calculated. Further, indications of probable scaling success (i.e., if items were grouped into a correct domain) were evaluated in accordance with Ware and Gandek [24] and defined as item-own correlation ≥ item-other-scale correlations using Pearson’s correlation coefficient. The opposite would indicate probable scaling failure.

#### Targeting

To evaluate whether the SCNS-P&C45-S could target the full variance within the sample, ceiling and floor effects and skewness were calculated. In accordance with Ware and Gandek and Hobert et al. [23, 24], ceiling and floor effects were considered as present if the proportion of response alternatives > 90% per item and skewness ranged outside -1 to 1.

#### Internal validity

Internal validity was evaluated in accordance with Hobart et al. [23] by determining whether domains measured similar but distinct aspects of the construct. Intercorrelations were calculated using Pearson’s correlation coefficient, with expected moderate correlation 0.30–0.70.

#### Construct validity

Convergent validity was evaluated based on a hypothesis by Chambers et al. [25] that family members who have high levels of anxiety also have more supportive care needs. As in previous evaluations of SCNS-P&C45 [3, 4, 15, 16], the relationship between the needs of support (total score of each domain) and anxiety measured by HADS (total score of the anxiety scale) was assessed using Spearman’s rank correlation coefficient. In accordance with Swinscow and Campbell [26], the threshold for acceptable correlation was set at ≥ 0.04. Level of significance was set to *p* < 0.05.

HADS is a 14-item scale measuring the number and severity of anxiety and depression symptoms on a four-point Likert scale [27]. The scale ranges from zero (*not at all*) to three (*very much*), with a total score ranging from 0 to 21. Higher scores indicate more psychological distress. Cronbach’s alpha values for HADS ranged between 0.71 and 0.90 for the total scale [28] and both subscales (anxiety and depression). The questionnaire has been validated in several populations [29], including persons diagnosed with cancer and their family members (e.g., Spinhoven [28] and Zigmond and Snaith [27].

#### Internal consistency

As per Hobart et al. [23], the internal consistency of the SCNS-P&C45-S and its allocated domains was evaluated using corrected item-to-total correlation, Cronbach’s alpha, and homogeneity. Cut-off criteria for satisfactory item-to-total correlations were > 0.4 in accordance with [24], for Cronbach’s alpha > 0.8, and for homogeneity > 0.3 [23].

### Ethical consideration

The study was conducted in accordance with the Declaration of Helsinki [30] and approved by the Swedish Ethical Review Authority (2020-04081, 2022-02144-02). The invited participants gave informed consent.

## Results

### Face and content validity

Face validity was considered good by both expert panels, although the nurses considered the questionnaire as too long. In contrast, the family members appreciated the comprehensive nature. Regarding content validity, S-CVI/UA (0.91) and the S-CVI/Ave (0.93) exceeded threshold values. Out of the 45 items, four were below the threshold values (0.66): Item 4, Item 24, Item 25, and Item 44. Due to the iterative approach of the translational process and since the I-CVI were just below cut off, no items were excluded.

Based on opinions of the expert panel of nurses, the following changes were made: “Partner and caregiver” was changed to “next of kin” since this concept in Swedish captures the meaning that the authors of the original SCNS-P&C45 aimed for with “partners and caregivers” – namely, a person involved in supporting the person diagnosed with cancer through the illness, who performs hands-on care and/or emotional support. Item 3, “Accessing information about support services…”, was changed to only “Accessing information about support for…” since “services” was not perceived as coherent in Swedish terminology. Item 8, “Accessing local health care services when needed”, was changed to “Accessing health care when needed” due to differences between Sweden and Australia in the organization of health care.

#### Cognitive interviews

Cognitive interviews with family members led to changing the heading “In the past month, what was your level of need for help with”, where “help” was replaced by “support” since the concept “help” was perceived too narrow in Swedish and non-logical in relation to the response alternatives. In addition, semantic suggestions were integrated to improve the understanding of the Swedish version. The wording in Item 43 “Explore your spiritual beliefs” was discussed but did not require revisions. Professor Girgis was informed about all conceptual changes.

### Psychometric evaluation, validity, and reliability

In total, 45 questionnaires were analysed, constituting a response rate of 31%. Participant characteristics are shown in Table 1. Psychometric properties of SCNS-P&C45-S are found in Table 2 and Table 3. Evaluations of construct validity are found in Table 4.

**Table 1:** The characteristics of the pre-test sample (*n* = 45)

Age	Mean (SD)	62.2	(15.4)
	Median (min max)	63	(26–86)
Gender, *n* (%)	Men	31	(69)
	Women	14	(31)
Relation, *n* (%)	Partner	31	(68)
	Child	11	(25)
	Friend	1	(2.3)
	Relative	2	(4.6)
Level of educational attainment, *n* (%)	Nine-year compulsory school	15	(11.1)
	Upper secondary school	10	(22.2)
	Higher education	27	(60)
	Missing data	3	(6.7)
Time since diagnose in months, *n* (%)	0–6 months	21	(46.7)
	6–12 months	14	(31.1)
	> 1 year	10	(22.2)
Time since surgery in months, *n* (%)	Prior to/no surgery	12	(16)
	0–6 months	16	(40)
	6–12	10	(22.2)
	> 1 year	5	(11.1)
	Missing data	3	(10.7)

**Table 2:** Psychometric properties of the translated and culturally adapted SCNS-P&C45-S questionnaire

Item*	Missing data *n* (%)	Item frequency distribution %					Item descriptive statistics		
		Not relevant	Satisfied	Low need	Moderate Need	High need	Mean	SD	Skewness
#1 Accessing information relevant to you as partner	0	22.7	36.4	9.1	20.5	11.4	1.61	1.35	0.460
#2 Accessing information about prognosis or likely outcome	0	9.1	45.5	9.1	13.6	22.7	1.95	1.38	0.419
#3 Accessing information about support for partners	0	27.3	31.8	11.4	15.9	11.4	1.51	1.37	0.558
#4 Accessing information about alternative therapies	0	18.2	43.2	9.1	15.9	13.6	1.64	1.33	0.591
#5 Accessing information on the person with cancer’s physical needs	0	22.7	36.4	9.1	22.7	9.1	1.59	1.31	0.433
#6 Accessing information about benefits and side-effects of treatments	0	13.6	40.9	15.9	15.9	13.6	1.75	1.28	0.494
#7 Obtaining the best medical care for the person diagnosed with cancer	0	18.2	45.5	11.4	0	25	1.68	1.46	0.728
#8 Accessing health care services	0	22.7	52.3	4.5	2.3	18.2	1.41	1.37	1.082
#9 Being involved in the person diagnosed with cancer’s care	0	31.8	38.6	9.1	9.1	11.4	1.30	1.32	0.941
#10 Having opportunities to discuss your concerns with the doctors	0	20.5	45.5	18.2	2.3	13.6	1.43	1.25	0.993
#11 Feeling confident that the doctors are coordinating care	0	18.2	47.7	6.8	6.8	18.2	1.58	1.38	0.797
#12 Ensuring an ongoing case manager to coordinate services for the person diangosed with cancer	0	18.2	50	9.1	4.5	18.2	1.55	1.36	0.899
#13 Making sure complaints are properly addressed	0	52.3	22.7	6.8	6.8	11.4	1.02	1.39	1.212
#14 Reducing stress in the person diagnosed with cancer’s life	0	22.7	29.5	22.7	13.6	11.4	1.61	1.30	0.440
#15 Looking after your own health	1 (2.3%)	29.5	34.1	15.9	4.5	15.9	1.43	1.39	0.804
#16 Obtaining adequate pain control	0	38.6	40.9	4.5	4.5	11.4	1.09	1.29	1.319
#17 Addressing fears about physical or mental deterioration	0	29.5	27.3	18.2	13.6	9.1	1.44	1.32	0.535
#18 Accessing information about the potential fertility problems	0	72.7	11.4	11.4	4.5	0	0.48	0.88	1.705
#19 Caring for the person diagnosed with cancer on a practical level	0	59.1	22.7	13.6	2.3	2.3	0.66	0.96	1.569
#20 Finding accessible hospital parking	0	50.0	29.5	9.1	4.5	6.8	0.89	1.19	1.457
#21 Adapting to changes to the person diagnosed with cancer’s working life or activities	0	36.4	29.5	18.2	11.4	4.5	1.18	1.19	0.769
#22 The impact that caring for the person diangosed with cancer has had on your working life or activities	0	47.7	18.2	20.5	9.1	4.5	1.05	1.22	0.878
#23 Finding out about financial support	1 (2.3%)	47.7	18.2	11.4	13.6	6.8	1.12	1.35	0.834
#24 Obtaining life and/or travel insurance for the person diagnosed with cancer	1 (2.3%)	56.8	13.6	18.2	2.3	6.8	0.86	1.23	1.262
#25 Accessing legal services	1 (2.3%)	77.3	0	13.6	6.8	0	0.56	1.18	1.912
#26 Communicating with the person diagnosed with cancer	1 (4.6%)	34.1	40.9	9.1	4.5	9.1	1.12	1.22	1.256
#27 Communicating with the family	0	34.1	43.2	6.8	2.3	13.6	1.18	1.32	1.250
#28 Getting support from your own family	0	38.6	43.2	11.4	2.3	2.3	0.84	0.90	1.273
#29 Talking to other persons who have cared for someone diagnosed with cancer	0	43.2	22.7	25	6.8	2.3	1.02	1.09	0.746
#30 Managing the topic of cancer in social situations	1 (2.3%)	50	27.3	18.2	2.3	2.3	0.80	0.98	1.213
#31 Managing concerns about recurrence	0	29.5	18.2	18.2	20.5	13.6	1.70	1.44	0.205
#32 The impact of cancer on your relationship	0	34.1	34.1	25	4.5	2.3	1.07	1.0	0.742
#33 Understanding the experience of the person diangosed with cancer	0	20.5	38.6	20.5	6.8	13.6	1.55	1.28	0.723
#34 Balancing your own needs with the needs of the person diagnosed with cancer	1 (2.3%)	34.1	34.1	15.9	9.1	4.5	1.14	1.15	0.857
#35 Adjusting to physical changes in the person diagnosed with cancer	0	47.7	34.1	15.9	0	2.3	0.75	0.89	1.349
#36 Addressing problems with your sex life	0	59.1	25	11.4	2.3	2.3	0.64	0.94	1.682
#37 Getting own emotional support	0	34.1	29.5	22.7	6.8	6.8	1.23	1.20	0.813
#38 Getting emotional support for your own family	0	50	22.7	20.5	0	6.8	0.91	1.16	1.315
#39 Managing feelings about death and dying	0	40.9	22.7	22.7	4.5	9.1	1.18	1.28	0.896
#40 Managing others not acknowledging the impact of caring for a person diagnosed with cancer on your life	0	40.9	27.3	27.3	0	4.5	1.00	1.06	0.992
#41 Coping with recovery not turning out as expected	1 (2.3%)	38.6	9.1	15.9	22.7	11.4	1.58	1.5	0.263
#42 Making decisions about your life	0	38.6	31.8	18.2	4.5	6.8	1.09	1.18	1.070
#43 Exploring your spiritual beliefs	0	72.7	15.9	6.8	0	4.5	0.48	0.98	2.502
#44 Finding meaning in the person diangosed with cancer’s illness	1 (2.3%)	54.5	11.4	20.5	2.3	6.8	0.90	1.25	1.163
#45 Having opportunities to participate in decision making	0	40.9	38.6	6.8	4.5	9.1	1.02	1.23	1.372

^*^Items are shortened. For full version see Girgis et al. [10]

**Table 3:** Psychometric evaluation of the SCNS-P&C45-S domains

Domains	Items	Mean	SD	Scale Mean if Item Deleted	Item-scale correlations							Cronbach’s Alpha if Item Deleted
					Own scale	Other Scales (Difference)						
						PEN		WSN		IN		
Domain 1 Health Care Service Needs (HCN)	Item 7	1.86	1.287	16.39	0.84	0.51	(0.33)^†^	0.68	(0.16)^†^	0.74	(0.10)^†^	0.95
	Item 8	1.64	1.183	16.61	0.69	0.31	(0.38)^†^	0.57	(0.12)^†^	0.44	(0.25)^†^	0.95
	Item 9	1.61	1.061	16.64	0.81	0.67	(0.15)^†^	0.81	(0)^††^	0.71	(− 0.10)^††^	0.95
	Item 10	1.64	1.059	16.61	0.79	0.68	(0.11)^†^	0.83	(− 0.14)^††^	0.81	(− 0.02)^††^	0.95
	Item 11	1.77	1.198	16.48	0.88	0.62	(0.26)^†^	0.76	(0.12)^†^	0.81	(0.07)^†^	0.94
	Item 12	1.73	1.188	16.52	0.91	0.55	(0.36)^†^	0.78	(0.13)^†^	0.72	(0.19)^†^	0.94
	Item 13	1.55	1.044	16.70	0.89	0.56	(0.33)^†^	0.81	(0.07)^†^	0.64	(0.25)^†^	0.94
	Item 14	1.84	1.055	16.41	0.71	0.56	(0.15)^†^	0.65	(0.06)^†^	0.67	(0.04)^†^	0.95
	Item 16	1.48	1.023	16.77	0.79	0.41	(0.38)^†^	0.73	(0.73)^†^	0.58	(0.21)^†^	0.95
	Item 17	1.75	1.014	16.50	0.81	0.56	(0.25)^†^	0.68	(0.13)^†^	0.69	(0.12)^†^	0.95
	Item 20	1.39	0.868	16.86	0.44	0.61	(− 0.25)^††^	0.47	(− 0.03)^††^	0.37	(0.07)^†^	0.96
	Cronbach’s alpha 0.95 Internal homogeneity 0.64				Probable Scaling Success 73%				Probable Scaling Failure 27%			

	Items	Mean	SD	Scale Mean if Item Deleted	Item-scale correlations							Cronbach’s Alpha if Item Deleted
					Own scale	Other scales (Difference)						
						WSN		IN		HCN		
Domain 2 Psychological and Emotional Needs (PEN)	Item 31	2.00	1.121	19.20	0.73	0.72	(0.08)^†^	0.81	(− 0.77)^††^	0.72	(0.01)^†^	0.91
	Item 32	1.41	0.693	19.80	0.64	0.46	(0.18)^†^	0.49	(0.15)^†^	0.51	(0.13)^†^	0.91
	Item 33	1.75	1.081	19.45	0.58	0.58	(0)^††^	0.73	(− 0.15)^††^	0.69	(− 0.11)^††^	0.91
	Item 34	1.50	0.849	19.70	0.70	0.56	(0.14)^†^	0.43	(0.27)^†^	0.43	(0.27)^†^	0.91
	Item 35	1.23	0.565	19.98	0.69	0.50	(0.19)^†^	0.55	(0.14)^†^	0.44	(0.44)^†^	0.91
	Item 36	1.23	0.605	19.98	0.54	0.17	(0.37)^†^	0.20	(0.34)^a^	0.12	(0.42)^a^	0.91
	Item 37	1.57	0.900	19.64	0.70	0.62	(0.08)^†^	0.51	(0.19)^†^	0.54	(0.16)^†^	0.91
	Item 38	1.41	0.816	19.80	0.45	0.65	(0.20)^†^	0.43	(0.02)^†^	0.49	(− 0.04)^††^	0.92
	Item 39	1.59	0.948	19.61	0.69	0.53	(0.16)^†^	0.51	(0.18)^†^	0.39	(0.30)^†^	0.91
	Item 40	1.41	0.726	19.80	0.75	0.63	(0.12)^†^	0.53	(0.22)^†^	0.52	(0.23)^†^	0.91
	Item 41	1.95	1.099	19.25	0.69	0.65	(0.04)^†^	0.79	(− 0.10)^††^	0.65	(0.04)^†^	0.91
	Item 42	1.48	0.876	19.73	0.69	0.61	(0.08)^†^	0.55	(0.14)^†^	0.51	(0.18)^†^	0.91
	Item 43	1.20	0.668	20.00	0.52	0.29	(0.23)^a^	0.13	(0.39)^a^	0.12	(0.40)^a^	0.91
	Item 44	1.48	0.849	19.73	0.59	0.41	(0.18)^†^	0.26	(0.33)^†^	0.22	(0.37)^†^	0.91
	Cronbach’s alpha 0.92 Internal homogeneity 0.45				Probable Scaling Success 79%				Probable Scaling Failure† 21%			

	Items	Mean	SD	Scale Mean if Item Deleted	Item-scale correlations				Cronbach’s Alpha if Item Deleted			
					Own scale	Other scales (Difference)						
						IN	HCN	PEN				
Domain 3 Work and Social Needs (WSN)	Item 21	1.55	0.875	8.55	0.52	0.54 (− 0.02)^††^	0.56 (0.04)^††^	0.67 (− 0.15)^††^	0.77			
	Item 22	1.52	0.849	8.57	0.39	0.39 (0)^††^	0.34 (0.05)^†^	0.61 (− 0.22)^††^	0.79			
	Item 26	1.48	0.952	8.61	0.71	0.68 (0.03)^†^	0.78 (− 0.07)^††^	0.47 (0.24)^†^	0.73			
	Item 27	1.52	1.067	8.57	0.54	0.49 (0.05)^†^	0.72 (− 0.18)^††^	0.37 (0.20)^†^	0.77			
	Item 28	1.27	0.660	8.82	0.39	0.47 (− 0.08)^††^	0.57 (− 0.18)^††^	0.31 (0.08)^†^	0.79			
	Item 29	1.45	0.730	8.64	0.65	0.53 (0.12)^†^	0.50 (0.15)^†^	0.66 (0.01)^††^	0.75			
	Item 30	1.30	0.632	8.80	0.55	0.47 (0.08)^†^	0.45 (0.10)^†^	0.50 (0.05)^†^	0.77			
	Cronbach’s alpha 0.80 Internal homogeneity 0.36				Probable Scaling Success 14%		Probable Scaling Failure† 86%					

	Items	Mean	SD	Scale Mean if Item Deleted	Item-scale correlations				Cronbach’s Alpha if Item Deleted			
					Own scale	Other scales (Difference)						
						IN	HCN	PEN				
Domain 4 Information Needs (IN)	Item 1	1.84	1.119	10.95	0.88	0.76 (0.12)^†^	0.57 (0.31)^†^	0.71 (0.17)^†^	0.91			
	Item 2	2.05	1.275	10.75	0.88	0.72 (0.16)^†^	0.60 (0.28)^†^	0.63 (0.25)^†^	0.91			
	Item 4	1.82	1.147	10.98	0.76	0.71 (0.05)^†^	0.51 (0.25)^†^	0.61 (0.15)^†^	0.92			
	Item 5	1.82	1.084	10.98	0.87	0.73 (0.14)^†^	0.56 (0.31)^†^	0.64 (0.23)^†^	0.91			
	Item 6	1.89	1.125	10.91	0.82	0.71 (0.11)^†^	0.57 (0.25)^†^	0.63 (0.19)^†^	0.91			
	Item 23	1.61	0.97	11.18	0.43	0.28 (0.15)^†^	0.46 (− 0.03)^b^	0.26 (0.17)^†^	0.95			
	Cronbach’s alpha 0.93 Internal homogeneity 0.64				Probable Scaling Success 86%		Probable Scaling Failure†14%					

^†^Probable Scaling Success = Item-own-scale correlation > item-other-scale correlation *p* < 0.05

^††^Probable Scaling Failure = Item-own-scale correlation ≤ item-other-scale-correlation *p* < 0.05

^a^Probable Scaling Success = Item-own-scale correlation > item-other-scale correlation *p* > 0.05

^b^Probable Scaling Failure = Item-own-scale correlation ≤ item-other-scale-correlation *p* > 0.05

**Table 4:** Correlations between SCNS-P&C45-S and HADS

	Health care service	Emotional and psychological	Work and social needs	Information needs
Anxiety	0.483**	0.602**	0.462**	0.412**

^**^Correlation is significant at the < 0.01 level

#### Data quality

In one case, the respondent completed < 50% of the questionnaire and thus, data from one person was excluded. Consequently, the scale score could be computed for 44 respondents (97.8%). Missing data per item were low and distributed across items (range 2.3–4.6%) (Table 2). All items were endorsed by 86% of the respondents (100% complete data). This indicates high data quality.

#### Scaling assumptions

Item means of the total SCNS-P&C45-S (five-point scale) ranged from 0.48 to 1.46, with responses distributed over all options (Table 2). Means across domains (four-point scale) ranged from 1.20 to 2.05 (Table 3). All items contributed substantially to their allocated domain (> 0.3), although correlations ranged from 0.32 to 0.91, indicating items can be summed. Probable scaling success ranged from 73 to 86% across domains (Table 3) apart from the Work and Social Needs domain, where probable scaling failure was 86%.

#### Targeting

Descriptive statistics (analysed for both four- and five-point scales) showed no ceiling or floor effect. Frequency distributions for item-response options were distributed across categories. Nonetheless, the item examination using the five-point scale showed three items deemed “not relevant” (response category 1) by over 70% of the participants: Item 18, Item 25, and Item 43. Further, the item-response frequency distribution was not symmetrical, a total of 19 items being highly right-skewed. These findings indicate potential problems in targeting. However, skewness of the scale was 0.69.

#### Internal validity

Correlations for internal validity ranged from 0.65 to 0.85 (mean 0.75), indicating redundancy.

#### Convergent validity

For convergent validity, there were, as expected, significant (*p* < 0.01) positive correlations between the total score of the domains and total score of the HADS Anxiety scale (0.41–0.60), supporting the construct validity of SCNS-P&C45-S. Correlations between the SCNS-P&C45-S domains and HADS are displayed in Table 4.

#### Internal consistency

The internal consistency of the SCNS-P&C45-S was 0.98 for the total scale. In evaluations of the internal consistency of the domains (Table 3), Cronbach’s alpha ranged from 0.80 to 0.95 across domains, apart from the Work and Social Needs domain (range 0.73–0.79). Average item-correlations ranged from 0.36 to 0.64, showing satisfactory internal consistency of the domains. Item-total correlations of the domains exceeded 0.3, yet a total of 20 items exceeded 0.7, indicating redundancy. In addition, for Item 20 and Item 23, the alpha value if the item was deleted exceeded Cronbach’s alpha of the domain, indicating unresolved problems with these items in their allocated domains.

## Discussion

The objective of this study was to translate, culturally adapt, and evaluate the psychometric properties of the SCNS-P&C45 by Girgis et al. [10]. As a final stage in an adaptation process, pre-testing among the target population was conducted [18]. Our pre-test showed that among Swedish family members of persons diagnosed with CRC, the SCNS-P&C45-S demonstrated promising validity and reliability. However, in the following, the questionnaires’ weaknesses will be discussed.

For targeting, some items (e.g., Item 18) were assessed “not relevant” by over 70% of the participants (Table 2), and 19 items were highly positively skewed. Item 18 showed floor effects in the original version, and in the Dutch version which was tested among partners of persons diagnosed with breast cancer [15] and ceiling effects among German family members of persons diagnosed with urological tumour, gastrointestinal tumour or lung cancer [2]. Consequently, the item was removed from all three versions. In contrast, no such effects were shown when the questionnaire was tested on a French population of family members of persons diagnosed with different cancers [3]. This indicates that despite the original generic intention of the questionnaire, items might not all be relevant for family members of all cancers. Thus, there is need for further investigation of targeting and relevance of items for family members of persons diagnosed with CRC, preferably using modern test theory since such examination according to Hobart and Cano [31] provide more precise answers to the appropriateness of items for a certain sample.

The evaluation of domains demonstrated probable scaling failure, indicating the domains as not functioning as intended in the current population. Previous psychometric evaluations have failed to replicate the overall factor structure of the original SCNS-P&C45 [2–4, 15, 16]. Therefore, confirming the factor structure of the SCNS-P&C45-S in a larger sample is warranted.

Despite some targeting issues, all items were kept in the current study, in contrast to the Dutch and German versions, since Hobart et al. [24] highlights that removing items which are included on a theoretical basis should be done with caution. For instance, Item 43 “Explore your spiritual beliefs” was assessed “not relevant” by 72.7% in the current study and exhibited a floor effect in the Dutch version [15]. Nonetheless, the literature highlights the importance of recognizing spiritual needs [1, 32], which was confirmed by religious family members in the expert panel. Despite Sweden being considered a secular society, it is still multicultural, and a questionnaire should be able to address the needs of a variety of respondents. Thus, it may be required to include an item in a scale to ensure content validity and measure the full range of a concept [24]. However, inadequate translation must also be considered. Item 43 was discussed extensively during the translation process, ending with consensus on the present form.

The internal consistency of the total scale (0.98) was comparable with the original and indicates satisfactory reliability. However, this should be interpreted with caution. First, it may be caused by the high number of items [33]. Second, Cronbach’s alpha being above 0.95, which was also found in the original survey and in the Dutch version [15], may indicate items measuring the same construct [33]. On the other hand, the alpha values across domains were 0.80–0.95 (apart from the Work and Social Needs), which could be considered satisfactory. However, the assumption of possible redundancy is supported by the mean correlation between scales being 0.75. Hence, exploring possible redundancy is also of relevance for future research. Perhaps, future studies could guide the development of a short form of the questionnaire, as has been done with the patient version [36–38] – especially since the expert panel of CRC specialist nurses considered the questionnaire to be too long for clinical practice. In addition, future studies using the SCNS-P&C-45 should consider that lengthy instruments such as this may not be a burden in isolation, as reported by the family members in the expert panel and reinforced by the low missing data. Yet, it may still be burdensome if having to undertake as one of many questionnaires.

### Methodological strengths and limitations

A strength of the current study is the systematic translation process, which was carefully monitored, recorded, and reported in accordance with the COSMIN checklist [17] to ensure transparency and traceability. Nevertheless, the study does not claim to have discovered all sematic or conceptual problems. Cognitive interviewing was conducted to discover such issues, and it ceased after seven interviews. Willis [20] recognized the possibility of adding more interviews to potentially yield substantial new insights. However, the second-round interviews in this study revealed no further questions or uncertainties, indicating the problems were recognized and resolved. In addition, the few missing items and lack of unused response categories [24] indicate a successful translation.

All participants in the expert panel were highly educated, and in the psychometric evaluation 11% were only compulsory educated. Despite Sweden could be considered a high-educated country this might lower the representativeness of the study and limit the conclusions that can be drawn, which points to the need for further evaluation of the questionnaire in a larger sample. Further, the response rate was low compared with previous studies (e.g., German 72.3%, Dutch 43%, and Chinese 98%). Reminders are significant for the response rate of a study [34]. However, in the current study, most of the family members were recruited indirectly via the person diagnosed with cancer; consequently, reminders were unfeasible. In addition, family members tend to focus on the person diagnosed with cancer [9, 35] and thus may not prioritize a questionnaire focusing on their own needs. In the German version, patients and family members were invited to answer the questionnaire in dyads, meaning that the patient answered the patient version and family members answered the partner and caregiver’s version. This might have had a positive impact on the willingness to answer. Despite the response-related limitation in the current study, no other way to invite family members of persons diagnosed with CRC was identified.

## Conclusion

The translated and adapted SCNS-P&C45-S questionnaire can be used to identify family members unmet needs of support throughout the CRC trajectory. In this study, the SCNS-P&C45-S showed promising validity and reliability among Swedish family members of persons diagnosed with CRC. Nonetheless, the pre-test revealed unsolved issues regarding relevance, targeting, and internal consistency, as well as a probable scaling failure. A more excessive evaluation of the questionnaire is warranted using a larger sample and including, for example, a confirmatory factor analysis as well as a psychometric evaluation using modern test theory. In addition, stability over time and responsiveness need to be evaluated.

## Data Availability

The datasets used and/or analysed during the current study are available from the corresponding author on reasonable request.

## Abbreviations

CRC: Colorectal cancer
HADSL: Hospital and anxiety scale
SCN: Supportive care needs
SCNS-P&C45: Supportive care needs survey partners and caregivers
SCNS-P&C45-S: Swedish supportive care needs survey partners and caregivers
I-CVI: Item content validity
S-CVI/Ave: Average scale content validity
S-CVI/UA: Universal agreement scale content validity
